# Metastatic pheochromocytoma and paraganglioma: signs and symptoms related to catecholamine secretion

**DOI:** 10.1007/s12672-021-00404-x

**Published:** 2021-03-19

**Authors:** Minghao Li, Christina Pamporaki, Stephanie M. J. Fliedner, Henri J. L. M. Timmers, Svenja Nölting, Felix Beuschlein, Aleksander Prejbisz, Hanna Remde, Mercedes Robledo, Stefan R. Bornstein, Jacques W. M. Lenders, Graeme Eisenhofer, Nicole Bechmann

**Affiliations:** 1 Department of Medicine III, University Hospital Carl Gustav Carus, Technische Universität Dresden, Dresden, Germany; 2 First Department of Medicine, University Medical Center Schleswig-Holstein, Lübeck, Germany; 3 Department of Internal Medicine, Radboud University Medical Center, Nijmegen, The Netherlands; 4 Medizinische Klinik Und Poliklinik IV, Klinikum der Ludwig-Maximilians-Universität München, Munich, Germany; 5 Department of Endocrinology, Diabetology and Clinical Nutrition, Universitätsspital Zürich, Zurich, Switzerland; 6 Department of Hypertension, Institute of Cardiology, Warsaw, Poland; 7 Division of Endocrinology and Diabetes, Department of Internal Medicine I, University Hospital of Würzburg, Würzburg, Germany; 8 Hereditary Endocrine Cancer Group, Spanish National Cancer Research Center and Centro de Investigación Biomédica en Red de Enfermedades Raras, Madrid, Spain; 9 Institute of Clinical Chemistry and Laboratory Medicine, University Hospital Carl Gustav Carus, Technische Universität Dresden, Dresden, Germany

**Keywords:** Pheochromocytoma, Paraganglioma, Metastatic, Signs, Symptoms, Catecholamines

## Abstract

**Background:**

The presence or future development of metastatic pheochromocytomas or paragangliomas (mPPGLs) can be difficult to diagnose or predict at initial presentation. Since production of catecholamines from mPPGLs is different from non-metastatic tumors (non-mPPGLs), this study aimed to clarify whether presenting catecholamine-related signs and symptoms (cSS) might also differ.

**Methods:**

The study included 249 patients, 43 with mPPGL and 206 with non-mPPGL. Clinical data at the time of biochemical diagnosis (i.e. at entry into the study) were used to generate a cumulative score of cSS for each patient.

**Results:**

Patients with mPPGL were significantly younger (43.3 ± 14 vs. 48.9 ± 16.1 years) and included a lower proportion of females (39.5% vs. 60.7%) than patients with non-mPPGLs. Frequencies of signs and symptoms did not differ between the two groups. Patients with mPPGLs had lower (P < 0.001) urinary excretion of epinephrine (3.5 (IQR, 1.9—6.5) µg/day) than those with non-mPPGLs (19.1 (IQR, 4.3—70.2) µg/day). There was no difference in urinary excretion of norepinephrine. In patients with mPPGLs a high cSS score was associated with high urinary excretion of norepinephrine and normetanephrine. In contrast, in patients with non-mPPGLs, a high cSS was associated with high urinary excretion of epinephrine and metanephrine.

**Conclusion:**

Although presenting signs and symptoms were associated with production of norepinephrine in patients with mPPGLs and of epinephrine in patients with non-mPPGLs, there were no differences in signs and symptoms between the two groups. Therefore, consideration of signs and symptoms does not appear helpful for distinguishing patients with and without mPPGLs.

**Supplementary Information:**

The online version contains supplementary material available at 10.1007/s12672-021-00404-x.

## Introduction

Pheochromocytoma and paraganglioma (PPGL) are rare neuroendocrine tumors arising from chromaffin cells of the adrenal medulla or extra-adrenal sympathetic or parasympathetic ganglia, respectively. Most patients with PPGLs can be cured surgically, but about 10–20% of patients present with or subsequently develop metastatic disease [1]. There are several factors that determine risk of developing metastases such as location and size of primary tumors and underlying genetic mutations, which also determine differences in catecholamine biochemical phenotypes [2].

Metastatic PPGLs (mPPGLs) produce mainly norepinephrine and/or dopamine and rarely express phenylethanolamine *N*-methytransferase (PNMT), the enzyme that converts norepinephrine to epinephrine [3, 4]. It is well established that mutations in succinate dehydrogenase B (*SDHB*) are associated with high risk of metastatic disease [5]. There is also a higher prevalence of mutations in genes causing activation of the pseudohypoxia pathway (cluster 1) relative to mutations in genes related to kinase signaling (cluster 2) in mPPGLs than in non-mPPGLs [6]. It has only been recently clarified that the association of cluster 1 mutations with metastatic disease is independent of the presence of *SDHB* mutations [7]. Furthermore, pro-metastatic behavior appears related to stabilization of hypoxia inducible factor 2 alpha (HIF2α), which blocks induction of PNMT and production of epinephrine, thereby explaining the link between genotype, biochemical phenotype and metastatic risk [3, 7].

The clinical signs and symptoms of PPGLs are highly variable and mainly relate to excessive tumoral production of catecholamines. Recurrent paroxysms of hypertension, headache, palpitations, diaphoresis and pallor represent the main clinical signs and symptoms. Anxiety, tremor, nausea, vomiting, and weight loss are also relevant to consider [8]. The highly heterogeneous clinical presentation in part reflects the episodic versus continuous nature of tumoral catecholamine secretion as well as the predominant type and amounts of catecholamine secreted by PPGLs [9, 10]. Epinephrine-producing tumors more often present with tremor, pallor and anxiety than tumors that do not produce epinephrine [8]. On the other hand tumors that produce exclusively norepinephrine tend to be associated more with sustained than episodic hypertension compared to epinephrine-producing tumors and this is in keeping with the more continuous than episodic nature of catecholamine secretion by the former than the latter tumors [11]. Since tumors in patients with mPPGLs secrete more norepinephrine and less epinephrine than those with non-mPPGLs [12], we hypothesized that patients of these two groups (metastatic versus non-metastatic) might differ in their presenting signs and symptoms and that this might assist in discriminating mPPGLs from non-mPPGLs. The objective of this study was therefore to evaluate signs and symptoms in patients with mPPGL and non-mPPGL. These patients were predominantly derived from a large cohort of patients enrolled into the prospective monoamine-producing tumor (PMT) study, the details of which have been described elsewhere [6, 13, 14].

## Subjects and methods

### Subjects

**Fig. 1:**
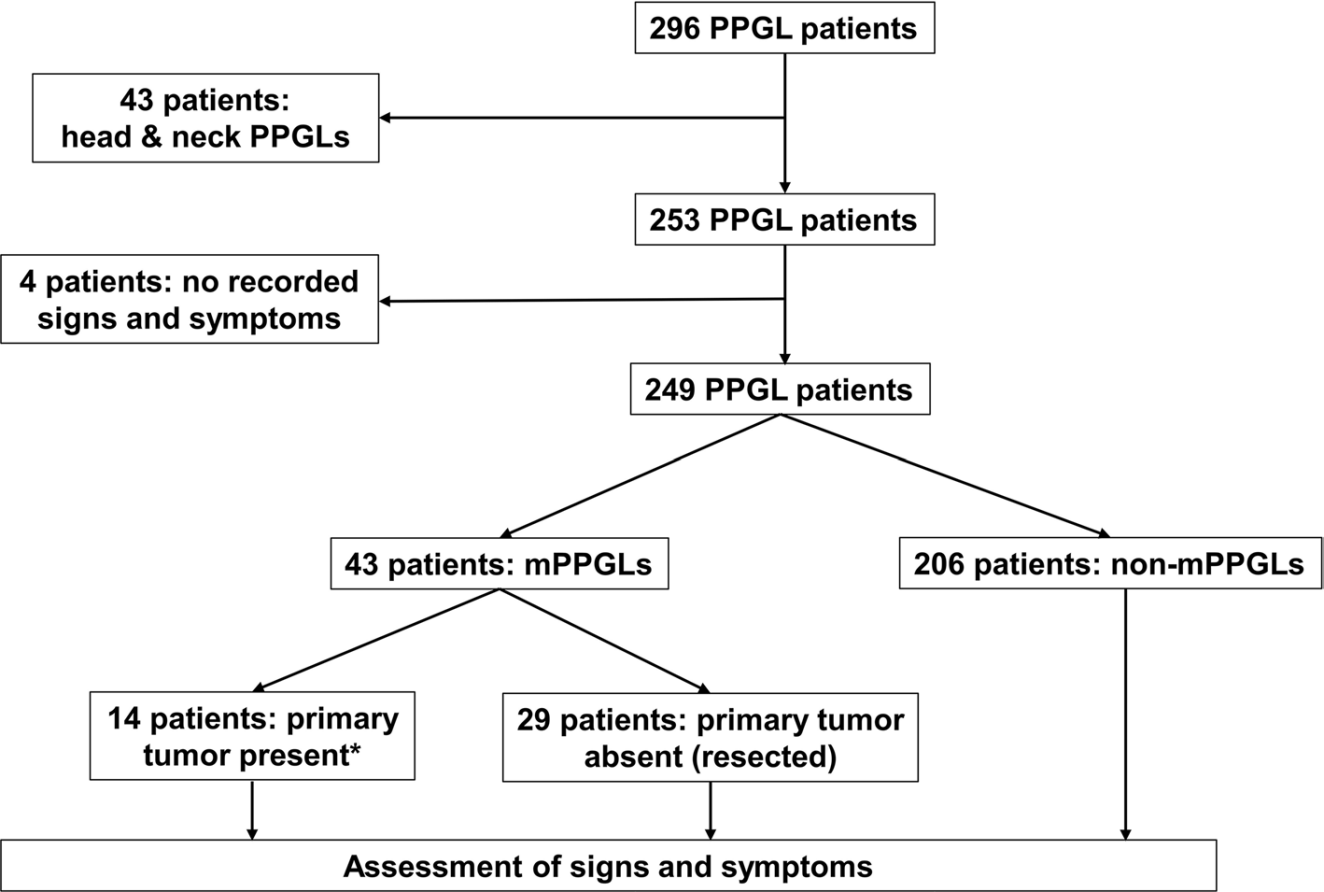
Flow chart showing the patients included and excluded in this study. mPPGL, metastatic pheochromocytoma/paraganglioma; non-mPPGL, non-metastatic pheochromocytoma/paraganglioma. *Three of 14 developed metastatic disease more than one year after study entry

Two hundred and eighty-nine patients with a proven PPGL were included from the PMT study cohort (https://pmt-study.pressor.org) as recruited between 2010 and 2019 at six tertiary care medical centers: 1. University Hospital Carl Gustav Carus Dresden, Germany; 2. University Medical Centre Schleswig–Holstein, Lübeck, Germany; 3. University Hospital of Munich, Germany; 4. University Hospital of Würzburg, Germany; 5. Radbound University Medical Centre, Nijmegen, the Netherlands; and 6. the Institute of Cardiology, Warsaw, Poland. Seven additional patients with PPGLs from Dresden were included after enrolment into a clinical trial (NCT03344016) that followed the PMT study. All patients provided signed informed consent under protocols approved by local ethics committees. Among the 296 patients, we excluded 43 with paragangliomas confined to the head and neck based on the usual asymptomatic presentation of patients with these tumors. Four patients were also excluded due to lack of recorded signs and symptoms (Fig. 1). Twenty-nine of the 43 patients with metastatic disease were identified with metastases after earlier resection of primary tumors. The other 14 patients had no previous history of PPGLs and had primary tumors with either evidence of metastatic disease at presentation or within one year after initial presentation (n = 11) or developed metastatic disease at a later time point on follow-up between 1 and 5 years after initial diagnosis of primary tumor (n = 3). The two subgroups of 29 and 14 patients with mPPGLs were respectively classified as patients with mPPGL in whom primary tumors were absent (resected) or present at the time of study entry.

### Diagnosis of metastatic disease

The 43 patients with mPPGLs were diagnosed with metastatic disease according to presence of metastases at locations where chromaffin cells are usually absent (e.g., bones, lungs, liver and lymph nodes), determined in most cases by ^123^I-MIBG or ^68^Ga-labeled DOTATATE functional imaging studies and usually supported by positive biochemical test results. Diagnosis of lymph node metastases required histopathological confirmation.

### Clinical data assessment

Signs and symptoms, covering a period of 30 days before study entry, were evaluated using a standardized questionnaire with data subsequently entered into electronic case report forms (eCRFs). Questions included the presence or absence of headaches, diaphoresis, palpitations, tremor, pallor, flushing, panic/anxiety, nausea/vomiting, weakness, abdominal pain, chest pain, paroxysmal hypertension and constipation. In addition, data on age at diagnosis of the primary tumor, sex, blood pressure (BP), heart rate (HR), body mass index (BMI) and antihypertensive medications were also recorded into eCRFs. The presence of hypertension was established by systolic BP above or equal to 140 mmHg or diastolic BP above or equal to 90 mmHg. Hypertension was also defined in patients with a history of high BP controlled by antihypertensive medications. A history of paroxysmal hypertension was assessed from patient notes and interviews.

### Biochemical measurements

Urinary free catecholamines and metanephrines were measured by liquid chromatography with tandem mass spectrometry (LC–MS/MS). Details including 24 h-urine collections, methods of measurement, and reference intervals have been described elsewhere [15, 16]. Results of catecholamines or metanephrines below the upper cut-off were defined as false negative results. Urine samples from 22 patients were not available.

### Genetic testing

Patients were initially tested for germline-mutations in established PPGL susceptibility genes at centers of recruitment or at the Hereditary Endocrine Cancer Group, CNIO, Madrid, Spain, using Sanger sequencing and/or next generation sequencing (NGS). Tumors with unknown genetic background were additionally tested for somatic mutations by customized panel-sequencing (Illumina, San Diego, CA) including coding exons and 20-bp of intronic flanking regions of known PPGL susceptibility genes including *RET*, *VHL*, *NF1*, *MAX*, *TMEM127*, *SDHA*, *SDHB*, *SDHC*, *SDHD*, *SDHAF2*, *MDH2*, *FH*, *EPAS1*, *HRAS*, *KIF1B*, *EGLN1*, *EGNL2*, *IDH1* and *IDH2*. Pathogenic mutations in *SDHA*, *SDHB*, *SDHC*, *SDHD*, *SDHAF2*, *VHL*, *FH*, *MDH2*, *EGLN1* and *EPAS1* were classified as cluster 1 PPGLs, while mutations in *TMEM127*, *MAX*, *RET*, *NF1* and *HRAS* were classified as cluster 2 PPGLs [17, 18]. In three patients mutation testing was not carried out.

### Data analysis

Differences in presentation of signs and symptoms in patients with and without metastatic disease were assessed by examination of each sign and symptom separately and according to a score system. For the latter, a cumulative clinical score for catecholamine-related signs and symptoms (cSS) was calculated for each patient, designating one point for each catecholamine-related sign or symptom (hypertension, headache, diaphoresis, palpitations, tremor, pallor, panic, nausea, classic triad, paroxysmal hypertension) being present or absent during the observation period. Based on the median cSS of all patients, patients were divided into low cSS score group (cSS < 4 points) and high cSS score group (cSS ≥ 4 points). In addition, differences in signs and symptoms and clinical features of mPPGL patients in presence or absence of the primary tumor (resected previously) were examined.

### Statistical analysis

IBM SPSS statistics 25 and SigmaPlot 12.5 (Systat Software GmnH, Ekrath, Germany) were used for data analysis. Levene’s test was used for testing of equality of variances. Continuous data that were normally distributed were presented as mean ± SDs and non-normally distributed data were expressed as medians and interquartile ranges (IQR). The Student-t test was used for normally distributed continuous data whereas Mann–Whitney test was used for non-normally distributed continuous data. Chi-square or the Fisher’s exact tests was used to compare categorical data. A linear regression model was used to examine relationships of cSS scores with urinary excretion of catecholamines and metanephrines.

## Results

### Clinical characteristics of patients with and without metastatic pheochromocytoma/paraganglioma

At the time of diagnosis of the primary tumor, patients with mPPGLs were younger (P = 0.034) than patients with non-mPPGLs (Table 1). Patients with mPPGLs also included less (P = 0.012) females than patients with non-mPPGLs. There were no differences in systolic and diastolic BP, heart rate, and BMI between both groups. Proportions of patients using antihypertensive drugs and numbers of antihypertensive medications used by patients were similar in both groups, but angiotensin-converting-enzyme inhibitors were taken more often by patients with non-mPPGL than mPPGL (27.6% vs. 11.6%, P = 0.032) (Additional file 1: Table S1). The prevalence of cluster 1 mutations was more than two fold higher (P < 0.001) in patients with mPPGLs than those with non-mPPGLs (Table1). In contrast, prevalence of cluster 2 mutations was threefold higher (P = 0.001) in patients with non-mPPGLs than mPPGLs. Mutations in known PPGL susceptibility genes were not found in 20 mPPGLs and 95 non-mPPGLs.

**Table 1:** Clinical characteristics of patients with metastatic and non-metastatic pheochromocytoma/paraganglioma

	mPPGL	non-mPPGL	P value
Patients, n	43	206	
Age at primary tumor diagnosis, mean ± SD (years)	43.3 ± 14	48.9 ± 16.1	0.034^b^
Systolic BP, mean ± SD (mmHg)	133.5 ± 16.9	136.4 ± 20.2	0.384^b^
Diastolic BP, mean ± SD (mmHg)	81.6 ± 12.6	83.5 ± 12	0.363^b^
Heart rate, mean ± SD (beats/min)	78.3 ± 15.4	78.4 ± 14.7	0.841^b^
BMI, mean ± SD (kg/m^2^)	24.2 ± 4.1	25.3 ± 4.6	0.155^b^
Females, n (%)	17 (39.5)	125 (60.7)	0.012^c^
Antihypertensive medication, n (%)	29 (67.4)	153 (74.3)	0.450^c^
Number of antihypertensive medications, median (IQR)	1 (0–3)	2 (0.75–3)	0.270^d^
Mutation, n (%)^a^			
Cluster 1	20 (46.2)	40 (19.7)	< 0.001^c^
Cluster 2	3 (10.3)	68 (33.5)	0.001^e^
No mutation found	20 (46.2)	95 (46.8)	1^c^

*mPPGL* metastatic pheochromocytoma/paraganglioma, *non-mPPGL* non-metastatic pheochromocytoma/paraganglioma, *BP* blood pressure, *BMI* body mass index, *IQR* interquartile ranges

^a^Pathogenic mutations in *SDHA*, *SDHB*, *SDHC*, *SDHD*, *SDHAF2*, *VHL*, *FH*, *MDH2*, *EGLN1/2* and *EPAS1* were classified as cluster 1 tumors, while mutations in *TMEM127*, *MAX*, *RET*, *NF1* and *HRAS* were classified as cluster 2 tumors

^b^Student-t test

^c^Chi-square test

^d^Mann–Whitney test

^e^Fisher’s test

There was no difference between both groups in the frequencies of hypertension or paroxysmal hypertension (Table 2). The most common symptom in patients with mPPGL was diaphoresis (48.8%), followed by weakness (46.5%), headaches (34.9%) and palpitations (32.6%). Palpitations were the most common symptom in patients with non-mPPGL (49%), followed by diaphoresis (45.6%), weakness (41.3%) and headaches (39.3%). There were no significant differences in clinical signs and symptoms between patients with non-mPPGLs and mPPGLs. In particular, the classic triad of palpitations, headache and diaphoresis did not differ. Taking into account the potential confounding influence of gender did not reveal any differences in signs and symptoms between patients with mPPGLs and non-mPPGLs (data not shown). The proportion of patients with a high cSS score in the mPPGL group was not different from that in the non-mPPGL group (37.2% vs. 49%, P = 0.181).

**Table 2:** Frequency of signs and symptoms in patients with metastatic and non-metastatic pheochromocytoma/paraganglioma

	mPPGL	non-mPPGL	P value
Patients, n	43	206	
Hypertension n (%)	33 (76.7)	170 (82.5)	0.390^c^
Paroxysmal hypertension, n (%)^a^	12 (27.9)	75 (37.1)	0.295^c^
Headaches, n (%)	15 (34.9)	81 (39.3)	0.611^c^
Diaphoresis, n (%)	21 (48.8)	94 (45.6)	0.739^c^
Palpitations, n (%)	14 (32.6)	101 (49)	0.064^c^
Tremor, n (%)	10 (23.3)	53 (25.7)	0.848^c^
Pallor, n (%)	9 (20.9)	57 (27.7)	0.449^c^
Flushing, n (%)	5 (11.6)	44 (21.4)	0.205^c^
Panic, n (%)	8 (18.6)	55 (26.7)	0.336^c^
Nausea, n (%)	6 (14)	47 (22.8)	0.225^c^
Weakness, n (%)	20 (46.5)	85 (41.3)	0.611^c^
Abdominal pain, n (%)	5 (11.6)	35 (17)	0.496^c^
Chest pain, n (%)	5 (11.6)	29 (14.1)	0.810^c^
Constipation, n (%)	6 (14)	29 (14.1)	1^c^
Classic triad, n (%)^b^	6 (14)	41 (19.9)	0.403^c^
cSS score, median (IQR)	3 (1–5)	3 (1.75–6)	0.111^d^
High cSS group, n (%)	16 (37.2)	101 (49)	0.181^c^

*mPPGL* metastatic pheochromocytoma/paraganglioma, *non-mPPGL* non-metastatic pheochromocytoma/paraganglioma, *cSS* cumulative catecholamine related signs and symptoms, *IQR* interquartile ranges

^a^Data of paroxysmal hypertension was not available in four non-mPPGLs

^b^Triad, the combined presence of headache, diaphoresis and palpitations

^c^Chi-square test

^d^Mann–Whitney test

### Urinary excretion of catecholamines and metanephrines

Patients with mPPGLs excreted less epinephrine than those with non-mPPGLs (3.5 (IQR, 1.9–6.5) vs. 19.1 (IQR, 4.3–70.2) µg/day, P < 0.001), but there were no differences in urinary norepinephrine excretion (Table 3). Similarly, patients with mPPGLs excreted less metanephrine than those with non-mPPGLs (19.3 (IQR, 10.9–41.4) vs. 94.2 (IQR, 22.3–347.5) µg/day, P < 0.001) and there was no significant difference in urinary normetanephrine. The proportion of false negative test results was significantly higher in patients with mPPGL than non-mPPGL and this applied to both catecholamines (48.6% vs. 17.9%, P < 0.001) and urinary metanephrines (16.2% vs. 4.7%, P = 0.021).

**Table 3:** Urinary excretion of catecholamines and metanephrines in patients with metastatic and non-metastatic pheochromocytoma/paraganglioma

	mPPGL	non-mPPGL	P value
Catecholamines, medians (IQR) (µg/day)			
Urine free norepinephrine	65.7 (31.2–299.4)	86.2 (40.6–211.4)	0.442^c^
Urine free epinephrine	3.5 (1.9–6.5)	19.1 (4.3–70.2)	< 0.001^c^
Metanephrines, medians (IQR) (µg/day)			
Urine free normetanephrine	280 (63.7–639.5)	223.7 (98.5–471.8)	0.9^c^
Urine free metanephrine	19.3 (10.9–41.4)	94.2 (22.3–347.5)	< 0.001^c^
False negative results, n (%)^a, b^			
Catecholamines	18 (48.6)	34 (17.9)	< 0.001^d^
Metanephrines	6 (16.2)	9 (4.7)	0.021^d^

*mPPGL* metastatic pheochromocytoma/paraganglioma, *non-mPPGL* non-metastatic pheochromocytoma/paraganglioma, *IQR* interquartile ranges

^a^Urine samples from six mPPGLs and 16 non-mPPGLs were not available

^b^Patients with catecholamines or metanephrines below the upper cut-off

^c^Mann–Whitney test

^d^Chi-square test

### Relationships between signs and symptoms with urinary catecholamines and metanephrines

**Fig. 2:**
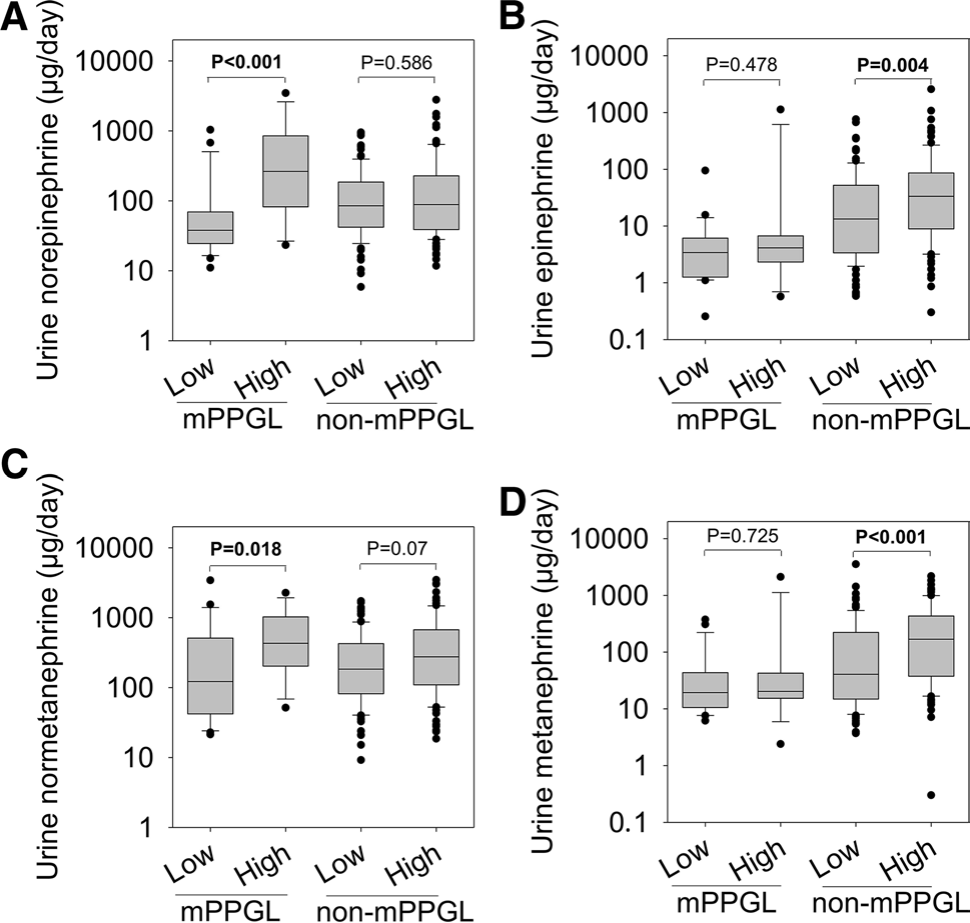
Urinary excretion of catecholamines and metanephrines in patients according to the cumulative score of signs and symptoms. *Low* low cumulative score of catecholamine related signs and symptoms, *High* high cumulative score of catecholamine related signs and symptoms, *mPPGL* metastatic pheochromocytoma/paraganglioma, *non-mPPGL* non-metastatic pheochromocytoma/paraganglioma

In patients with mPPGLs and a high cSS score, urinary excretion of norepinephrine was considerably higher (P < 0.001) than in patients with a low cSS score (Fig. 2a). Also, the cSS score of patients with mPPGLs was positively related (R^2^ = 0.139, P = 0.023) with the urinary excretion of norepinephrine (Additional file 1: Figure S1A). Patients with a high cSS score also showed higher (P = 0.018) excretion of normetanephrine than those with a low cSS score (Fig. 2c), but the cSS score did not correlate with the urinary excretion of normetanephrine (Additional file 1: Figure S1E). In contrast, urinary excretion of epinephrine and metanephrine did not differ between patients with mPPGLs who had high versus low cSS scores (Fig. 2b, d) and showed no significant relationships with the cSS scores (Additional file 1: Figure S1C and G).

Among patients with non-mPPGLs, urinary excretion of epinephrine was higher (P = 0.004) in those with high cSS scores than with low cSS scores (Fig. 2b), and showed a positive relationship (R^2^ = 0.034, P = 0.01) with cSS score (Additional file 1: Figure S1D). Similarly, urinary excretion of metanephrine was significantly higher in patients with a high cSS score (P = 0.004) (Fig. 2d) and showed a positive relationship (R^2^ = 0.048, P = 0.002) with cSS score (Additional file 1: Figure S1H). Urinary excretion of norepinephrine and normetanephrine in patients with non-mPPGLs and a high cSS score were not different from those with a low cSS score (Fig. 2a, c), but showed weak positive relationships (norepinephrine: R^2^ = 0.03, P = 0.017; normetanephrine: R^2^ = 0.043, P = 0.004) with cSS scores (Additional file 1: Figure S1B and F).

### Differences in patients with mPPGLs in presence or absence (resected) of the primary tumor

Patients with mPPGLs in whom removal of the primary tumor was performed before study entry were younger than those in whom the primary tumor was present at study entry and biochemical testing (40.3 ± 14.2 vs. 49.5 ± 11.6 years, P = 0.041) (Table 4). Previous resection of the primary tumor in patients with mPPGLs was not associated with differences in BP, HR, BMI, gender, medication use or nature of gene mutations compared to patients with presence of primary tumors at study entry (Table 4). In presence of the primary tumor at study entry, patients with mPPGL were more prone to abdominal pain than mPPGL patients in whom the primary tumor had already been resected (28.6% vs. 3.4%, P = 0.032) (Table 4). Catecholamine related signs and symptoms did not differ between the two groups (Table 4). The presence of the primary tumor was also not associated with any differences in the urinary excretion of catecholamines and metanephrines in patients with mPPGLs (Additional file 1: Table S2).

**Table 4:** Clinical characteristics and signs and symptoms in mPPGLs in absence or presence of the primary tumor

	mPPGL, primary tumor present^a^	mPPGL, primary tumor absent (resected)	P value
Patients, n	14	29	
Age at primary tumor diagnosis, mean ± SD (years)	49.5 ± 11.6	40.3 ± 14.2	0.041^c^
Systolic BP, mean ± SD (mmHg)	132.6 ± 12.9	134 ± 18.8	0.144^c^
Diastolic BP, mean ± SD (mmHg)	81.7 ± 8.7	81.6 ± 14.2	0.092^c^
Heart rate, mean ± SD (beats/min)	84.4 ± 17.1	76.3 ± 14.1	0.333^c^
BMI, mean ± SD (kg/m^2^)	22.7 ± 3.3	24.9 ± 4.2	0.109^c^
Females, n (%)	5 (35.7)	12 (41.4)	0.753^d^
Antihypertensive medication, n (%)	11 (64.7)	18 (69.2)	1^d^
Number of antihypertensive medications, median (IQR)	1 (0–3.25)	1 (0–2.5)	0.715^e^
Mutation, n (%)^b^			
Cluster 1	9 (64.3)	11 (37.9)	0.191^d^
Cluster 2	1 (7.1)	2 (6.9)	1^f^
No mutation found	4 (28.6)	16 (55.2)	0.119^d^
Signs and symptoms			
Hypertension n (%)	12 (85.7)	21 (72.4)	0.456^d^
Paroxysmal hypertension, n (%)	4 (28.6)	8 (27.6)	1^d^
Headaches, n (%)	5 (35.7)	10 (34.5)	1^d^
Diaphoresis n (%)	8 (57.1)	13 (44.8)	0.526^d^
Palpitations, n (%)	6 (42.9)	8 (27.6)	0.488^d^
Tremor, n (%)	4 (28.6)	6 (20.7)	0.704^d^
Pallor, n (%)	3 (21.4)	6 (20.7)	1^f^
Flushing, n (%)	1 (7.1)	4 (13.8)	0.655^f^
Panic, n (%)	3 (21.4)	5 (17.2)	1^f^
Nausea, n (%)	2 (14.3)	4 (13.8)	1^f^
Weakness, n (%)	7 (50)	13 (44.8)	1^d^
Abdominal pain, n (%)	4 (28.6)	1 (3.4)	0.032^f^
Chest pain, n (%)	2 (14.3)	3 (10.3)	1^f^
Constipation, n (%)	1 (7.1)	5 (17.2)	0.654^f^
Classic triad, n (%)	3 (21.4)	3 (10.3)	0.373^f^
cSS score, median (IQR)	3 (1–5)	2 (1–4)	0.345^e^
High cSS group, n (%)	6 (42.9)	10 (34.5)	0.340^d^

*mPPGL* metastatic pheochromocytoma/paraganglioma, *BP* blood pressure, *BMI* body mass index, *cSS* cumulative catecholamine related signs and symptoms, *IQR* interquartile ranges

^a^Three of 14 develop metastatic disease one year later after study entry

^b^Pathogenic mutation in *SDHA*, *SDHB*, *SDHC*, *SDHD*, *SDHAF2*, *VHL*, *FH*, *MDH2*, *EGLN1/2* and *EPAS1* were classified as cluster 1, while mutation in *TMEM127*, *MAX*, *RET*, *NF1* and *HRAS* were classified as cluster 2

^c^Student-t test

^d^Chi-square test

^e^Mann–Whitney test

^f^Fisher’s test

## Discussion

In this study we found no differences in signs and symptoms associated with catecholamine excess between patients with and without mPPGLs. This applied not only to singular signs and symptoms but also to the cumulative cSS score. A high burden of signs and symptoms was associated with high urinary excretion of norepinephrine and normetanephrine in patients with mPPGLs, whereas in patients without metastases the association was stronger for urinary epinephrine and metanephrine. This difference presumably reflects lower tumoral epinephrine production in patients with than without mPPGLs combined with lack of significant differences in urinary excretion of norepinephrine.

Catecholamines are responsible for signs and symptoms while the metanephrines, as metabolites of catecholamines, are biologically inactive. Nevertheless, the metabolites are commonly used for diagnosis of PPGLs and are also more informative than the parent catecholamines for determining differences in tumoral catecholamine biochemical characteristics. Such characteristics are useful for predicting tumor location and size as well as underlying genetic mutations and presence of metastases [19, 20]. PPGLs that produce epinephrine present with different constellations of symptoms than PPGLs that produce norepinephrine [8]. It is also well established that non-mPPGLs produce more epinephrine than mPPGLs [21].

Although we confirmed that non-mPPGLs produced more epinephrine than mPPGLs, we were unable to establish any associated differences in signs and symptoms. This may be explained in several ways. First, differences in catecholamine production between patients with and without mPPGLs were highly variable and for epinephrine the differences may have been insufficient to cause catecholamine-specific differences in signs and symptoms. Second, although signs and symptoms were recorded systematically and included provision for establishing frequency, duration, and association with other symptoms, the latter were inconsistently completed within eCRFs. Finally, with longer follow-up, some patients classified with non-mPPGLs may in future be classified as mPPGLs.

As expected, patients with mPPGLs had a higher prevalence of genetic mutations related to the pseudohypoxia pathway (cluster 1) than those with non-mPPGLs. Patients with cluster 1 tumors show a more immature biochemical phenotype than those with cluster 2 tumors; this includes lack of epinephrine production and lower tumor contents of catecholamines in the former than the latter tumors [9]. Extra-adrenal paragangliomas and PPGLs in patients with *SDHB* mutations show particularly low contents of catecholamines compared to other catecholamine-producing tumors [4, 22]. Since extra-adrenal locations and *SDHB* mutations are relatively common in metastatic PPGLs, the above differences likely explain why patients with mPPGLs more often presented with false-negative results for catecholamines and metanephrines than patients with non-mPPGLs. The much larger proportion of false-negative results for catecholamines in patients with mPPGLs would also be expected to contribute to the considerable variance in signs and symptoms in patients with mPPGLs, thereby masking differences between patient groups with larger increases in tumoral catecholamine secretion.

Previous reports examining differences in clinical presentation of patients with and without metastatic PPGLs have not revealed consistent findings or examined differences in relation to catecholamine secretion [23, 24]. Mashaal et al. reported that the proportion of symptomatic patients with and without metastatic PPGLs was not different [13]; however, a detailed comparative description of presenting signs and symptoms between two groups was missing. Several other studies reported on signs and symptoms in patients with mPPGLs, but comparative data including an appropriate group of patients without metastatic disease was also lacking [6, 14, 25]. Some studies, focusing on specific symptoms, found a higher proportion of patients with mPPGLs complaining of severe constipation compared to those without metastatic disease [26–28]. We could not confirm any difference in constipation between groups, though this complaint is likely associated with larger tumor burden and secretion of norepinephrine than in most of our patients.

The clinical implication of our findings is that catecholamine-related signs and symptoms in patients with PPGLs cannot be used to predict the presence of mPPGL. Even a cumulative scoring system seems not to overcome this shortfall of clinical symptomatology.

A particular strength of this study is the prospective assessment of signs and symptoms in the majority of patients. In addition, signs and symptoms were recorded at the time of biochemical measurements, enabling an assessment of the relationship between clinical and biochemical phenotype. There are also several limitations. It was not possible to assess tumor burden as one of the determinants of catecholamine production in patients with metastatic disease where this would require imaging analyses of all lesions. Since a previous study reported that female patients with PPGLs demonstrate more self-reported signs and symptoms than male patients [29] and since there were proportionally more males than females with metastatic disease, this might have had a confounding influence on interpretations. Nevertheless, with multivariable analysis to account for this potential confounder we were unable to establish any differences in signs and symptoms between the two groups.

In conclusion, despite clear differences in the catecholamine secretion patterns between patients with and without metastatic PPGLs, this study demonstrates that presenting signs and symptoms do not appear to differ between the two groups. Therefore, signs and symptoms cannot be used for identification of patients with PPGLs, who either have metastases at presentation or who are prone to future metastatic development.

## Supplementary Information

Below is the link to the electronic supplementary material.

**Additional file 1: Figure S1.** Correlations between urinary excretion of catecholamines and metanephrines with scores of cumulative catecholamine related signs and symptoms. **Table S1.** The number of patients with and without metastatic pheochromocytoma/paraganglioma using specific antihypertensive medications. **Table S2.** Urinary excretions of catecholamines and metanephrines in mPPGLs with primary tumor present versus absent.

## Data Availability

https://pmt-study.pressor.org.
