# Supplementary material for: Metastatic pheochromocytoma and paraganglioma: signs and symptoms related to catecholamine secretion
Source: Discov Oncol. 2021 Mar 19;12:9. doi: 10.1007/s12672-021-00404-x (PMC8777503; doi:10.1007/s12672-021-00404-x)
Supplement: Supplementary file 1 — Additional file 1: Figure S1. Correlations between urinary excretion of catecholamines and metanephrines with scores of cumulative catecholamine related signs and symptoms. Table S1. The number of patients with and without metastatic pheochromocytoma/paraganglioma using specific antihypertensive medications. Table S2. Urinary excretions of catecholamines and metanephrines in mPPGLs with primary tumor present versus absent. [file 12672_2021_404_MOESM1_ESM.docx]

**Additional file 1**

**Metastatic Pheochromocytoma and Paraganglioma:**

**Signs and Symptoms Related to Catecholamine Secretion**

Minghao Li^1^, Christina Pamporaki^1^, Stephanie M. J. Fliedner^2^, Henri J. L. M. Timmers^3^, Svenja Nölting^4,5^, Felix Beuschlein^4,5^, Aleksander Prejbisz^6^, Hanna Remde^7^, Mercedes Robledo^8^, Stefan R. Bornstein^1^, Jacques W. M. Lenders^1,3^, Graeme Eisenhofer^1,9^, Nicole Bechmann^1,9,^*

^1^Department of Medicine III, University Hospital Carl Gustav Carus, Technische Universität Dresden, Dresden, Germany

^2^First Department of Medicine, University Medical Center Schleswig-Holstein, Lübeck, Germany

^3^Department of Internal Medicine, Radboud University Medical Center, Nijmegen, The Netherlands

^4^Medizinische Klinik und Poliklinik IV, Klinikum der Ludwig-Maximilians-Universität München, Munich, Germany

^5^Department of Endocrinology, Diabetology and Clinical Nutrition, Universitätsspital Zürich, Zurich, Switzerland

^6^Department of Hypertension, Institute of Cardiology, Warsaw, Poland

^7^Division of Endocrinology and Diabetes, Department of Internal Medicine I, University Hospital of Würzburg, Würzburg, Germany

^8^Hereditary Endocrine Cancer Group, Spanish National Cancer Research Center and Centro de Investigación Biomédica en Red de Enfermedades Raras, Madrid, Spain

^9^Institute of Clinical Chemistry and Laboratory Medicine, University Hospital Carl Gustav Carus, Technische Universität Dresden, Dresden, Germany


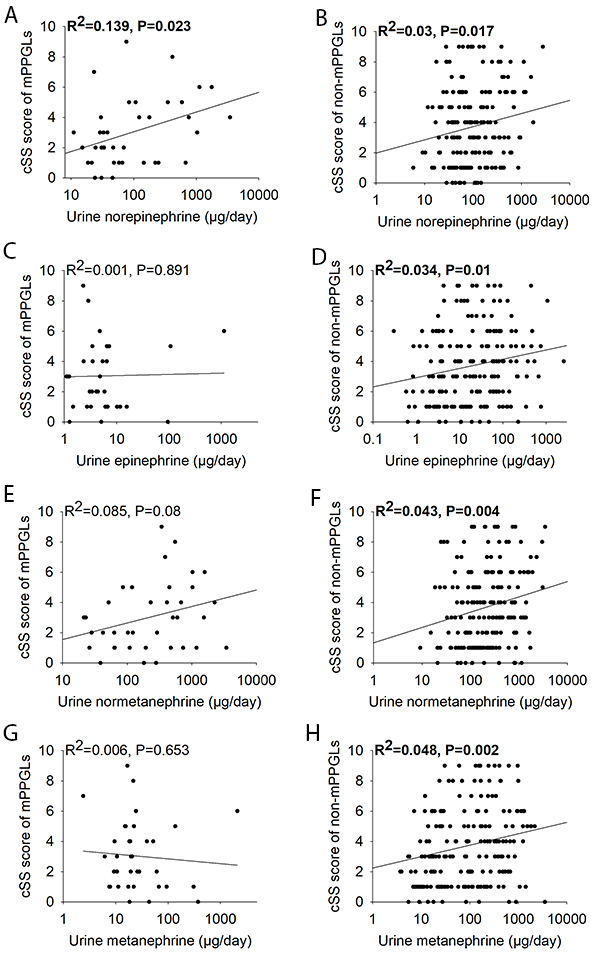


**Figure S1. Correlations between urinary excretion of catecholamines and metanephrines with scores of cumulative catecholamine related signs and symptoms.**

Panels A, C, E and G, the correlations between cSS score with the urinary excretion of catecholaminesand metanephrines respectively in mPPGL patients; Panels B, D, F and H, the correlations between the cSS scores with the urinary excretion of catecholamines and metanephrines respectively in the non-mPPGL patients. Linear regression was used to build the models. cSS score, cumulative score of catecholamine related signs and symptoms; mPPGLs, metastatic pheochromocytomas/paragangliomas; non-mPPGLs, non-metastatic pheochromocytomas/paragangliomas.

**Table S1:** The number of patient**s** with and without metastatic pheochromocytoma/paraganglioma using specific antihypertensive medications.

| Medication | mPPGL | non-mPPGL | P value |
| --- | --- | --- | --- |
| Diuretics, n (%) | 6 (14) | 29 (14.3) | 1^a^ |
| Ca^2+^ blockers, n (%) | 9 (20.9) | 56 (27.6) | 0.449^a^ |
| Beta-blockers, n (%) | 15 (34.9) | 79 (38.9) | 0.731^a^ |
| Alpha-blockers, n (%) | 15 (34.9) | 56 (27.6) | 0.354^a^ |
| ACE-inhibitors, n (%) | 5 (11.6) | 56 (27.6) | 0.032^a^ |
| AT-II receptor blockers, n (%) | 5 (11.6) | 25 (12.3) | 1^a^ |
| Renin Inhibitors, n (%) | 0 | 1 (0.5) | 1^b^ |
| Central alpha -2 agonists, n (%) | 3 (7) | 3 (1.5) | 0.066^b^ |
| MRAs, n (%) | 1 (2.3) | 9 (4.4) | 0.699^b^ |
| RAS inhibitors, n (%) | 10 (23.3) | 76 (37.8) | 0.08^a^ |

^a^ Chi-square test, ^b^ Fisher’s test.

ACE, angiotensin-converting-enzyme; AT-II, angiotensin II; MRAs, mineralocorticoid receptor antagonists; RAS Renin-angiotensin-aldosterone system.

**Table S2:** Urinary excretions of catecholamines and metanephrines in mPPGLs with primary tumor present versus absent.

|  | mPPGL with primary tumor present^*^ | mPPGL with primary tumor absent (resected) | P value |
| --- | --- | --- | --- |
| Catecholamines, medians (IQR) (µg/day) |  |  |  |
| - Urine free norepinephrine | 122.3 (38 – 350.3) | 47.7 (24.3 – 236.8) | 0.120^a^ |
| - Urine free epinephrine | 5.4 (2.3 – 10.2) | 3.3 (1.4 – 5.9) | 0.271^a^ |
| Metanephrines, medians (IQR) (µg/day) |  |  |  |
| - Urine free normetanephrine | 412 (193.5 – 733.7) | 151.8 (60 – 617.8) | 0.196^a^ |
| - Urine free metanephrine | 18.9 (15.5 – 43.4) | 19.9 (10.3 – 42.6) | 0.806^a^ |
| False negative results, n (%)^$, #^ |  |  |  |
| - Catecholamines | 3 (27.3) | 15 (57.7) | 0.151^b^ |
| - Metanephrines | 1 (9.1) | 5 (19.2) | 0.646^b^ |

^*^ Three of 14 develop metastatic disease one year later after study entry. ^$^ Urine samples from three mPPGLs with primary tumor present and three mPPGLs with absent primary tumor (resected) were not available. ^#^ Patients with catecholamines or metanephrines below the upper cut-off.
^a^ Mann-Whitney test, ^b^ Fisher’s test.
mPPGL, metastatic pheochromocytoma/paraganglioma; IQR, interquartile ranges.
